# In Vitro Immunostimulating Activity of Sulfated Polysaccharides from *Caulerpa cupressoides* Var. *Flabellata*

**DOI:** 10.3390/md17020105

**Published:** 2019-02-09

**Authors:** Jefferson da Silva Barbosa, Mariana Santana Santos Pereira Costa, Luciana Fentanes Moura de Melo, Mayara Jane Campos de Medeiros, Daniel de Lima Pontes, Katia Castanho Scortecci, Hugo Alexandre Oliveira Rocha

**Affiliations:** 1 Laboratório de Biotecnologia de Polímeros Naturais (BIOPOL), Departamento de Bioquímica, Centro de Biociências, Universidade Federal do Rio Grande do Norte (UFRN), Natal, Rio Grande do Norte 59078-970, Brazil; jefferson.barbosa@ifrn.edu.br (J.d.S.B.); lucianafentanes@gmail.com (L.F.M.d.M.); 2 Programa de Pós-Graduação em Ciências da Saúde, Universidade Federal do Rio Grande do Norte (UFRN), Natal, Rio Grande do Norte 59012-570, Brazil; 3 Instituto Federal de Educação, Ciência e Tecnologia do Rio Grande do Norte (IFRN), São Gonçalo do Amarante, Rio Grande do Norte 59291-727, Brazil; 4 Instituto Federal de Educação, Ciência e Tecnologia do Rio Grande do Norte (IFRN), João Câmara, Rio Grande do Norte 59550-000, Brazil; mariana.costa@ifrn.edu.br; 5 Laboratório de Química de Coordenação e Polímeros (LQCPol), Instituto de Química, Universidade Federal do Rio Grande do Norte (UFRN), Natal, Rio Grande do Norte 59078-970, Brazil; mayarajane20049@hotmail.com (M.J.C.d.M.); pontesdl@yahoo.com (D.d.L.P.); 6 Laboratório de Transformação de Plantas e Análise em Microscopia, Departamento de Biologia Celular e Genética, Centro de Biociências, Universidade Federal do Rio Grande do Norte, Natal, Rio Grande do Norte 59078-970, Brazil; kacscort@yahoo.com

**Keywords:** sulfated polysaccharides, immunostimulatory activity, macrophages, inflammatory mediators

## Abstract

Green seaweeds are rich sources of sulfated polysaccharides (SPs) with potential biomedical and nutraceutical applications. The aim of this work was to evaluate the immunostimulatory activity of SPs from the seaweed, *Caulerpa cupressoides* var. *flabellata* on murine RAW 264.7 macrophages. SPs were evaluated for their ability to modify cell viability and to stimulate the production of inflammatory mediators, such as nitric oxide (NO), intracellular reactive oxygen species (ROS), and cytokines. Additionally, their effect on inducible nitric oxide synthase (iNOS) and cyclooxygenase 2 (COX-2) gene expression was investigated. The results showed that SPs were not cytotoxic and were able to increase in the production of NO, ROS and the cytokines, tumor necrosis factor alpha (TNF-α) and interleukin 6 (IL-6). It was also observed that treatment with SPs increased iNOS and COX-2 gene expression. Together, these results indicate that *C. cupressoides* var. *flabellata* SPs have strong immunostimulatory activity, with potential biomedical applications.

## 1. Introduction

Seaweeds are sources of bioactive compounds with potential biotechnological applications and may potentially be exploited as functional ingredients for human health [1,2]. Among these compounds, sulfated polysaccharides (SPs) are the main components of the cell wall of seaweeds and represent an important structural component of these organisms [3,4]. These SPs exhibit many beneficial biological activities such as anticoagulant, antiviral, antioxidative, anticancer and immunomodulating activities [5]. Therefore, marine algae derived SPs have great potential for further development as products in nutraceutical, pharmaceutical and cosmeceutical areas [6].

The discovery and evaluation of the biological activities of SPs has emerged as a promising research field. In the last few decades, several studies have reported that SPs are able to interact with surface receptors of animal cells and participate in several processes like cell recognition, cell adhesion, and the regulation of cellular processes of biomedical interest [7], including those involved in modulating the immune response [8,9,10]. Molecules with immunostimulatory effects, such as SPs, may become interesting therapeutic agents because they are capable of potentiating natural defenses against immunosuppressive conditions, as in individuals undergoing cancer treatment, as well in fighting against infections caused by microorganisms [11,12,13].

SPs have, among other characteristics, low toxicity and as a result, cause few adverse effects. These compounds stimulate different types of immune system cells, in vitro and *in vivo*, to produce and secrete molecules with immunostimulatory effects [4,14,15]. Hence, they have therapeutic potential as adjuvants for the treatment of immunological disorders.

Macrophages are important effector cells of innate immunity, but they also help in the initiation and propagation of the acquired immune response. Therefore, the search for agents that modulate the activity of macrophages is of great interest in biomedical research [16]. Studies of SPs obtained from different natural sources have shown that these molecules stimulate macrophages to increase their cytotoxic and immunomodulatory activities, through the production of nitric oxide (NO), reactive oxygen species (ROS), prostaglandins, the production and secretion of cytokines [17,18,19]. Often these inflammatory mediators are studied by quantification in cell culture supernatants or by analyzing their gene expression, such as for the enzymes, inducible nitric oxide synthase (iNOS) and cyclooxygenase-2 (COX-2) [20,21,22].

The seaweed, *Caulerpa cupressoides* var. *flabellata*, synthesizes four distinct SPs populations, CCB-F0.3, CCB-F0.5, CCB-F1.0, and CCB-F2.0 [23]. CCB-F0.3 presents the main polysaccharide with a molecular weight of 155 kDa, consisting of the monosaccharides, galactose, glucose, mannose, and xylose in a molar ratio of 1.0:0.1:0.2:0.1. CCB-F0.5 has polysaccharide of 130 kDa, consisting of galactose and mannose in a molar ratio of 1.0:0.1 and traces of xylose. CCB-F1.0 presents as a 155 kDa polysaccharide with galactose, mannose, and xylose in a molar ratio of 1.0:0.1:0.6 and traces of glucose and rhamnose. CCB-F2.0 presents as a 170 kDa polysaccharide with galactose, glucose, mannose, xylose, rhamnose, and fucose in a molar ratio of 1.0:0.6:1.8:1.0:0.5:1.0. Although previous studies have characterized the antiproliferative, anticoagulant, and antioxidant activities of SPs from *C. cupressoides* var. *flabellata* [23,24], their immunostimulatory properties remain unknown. Therefore, the aim of this work was to evaluate the immunostimulatory potential of theirs SPs on murine macrophage RAW 264.7 cell line.

## 2. Results and Discussion

Using the process of extracting *C. cupressoides* SPs described by Costa et al. [23] and in the Materials and Methods section of this paper, four SPs-rich fractions were obtained that were dominated by CCB-F0.3, CCB-F0.5, CCB-F1.0, and CCB-F2.0. These fractions were evaluated by gel electrophoresis and infrared spectroscopy to confirm their identity.

### 2.1. Agarose Gel Electrophoresis in 1,3-diamino Propane Acetate Buffer (PDA)

**Figure 1:**
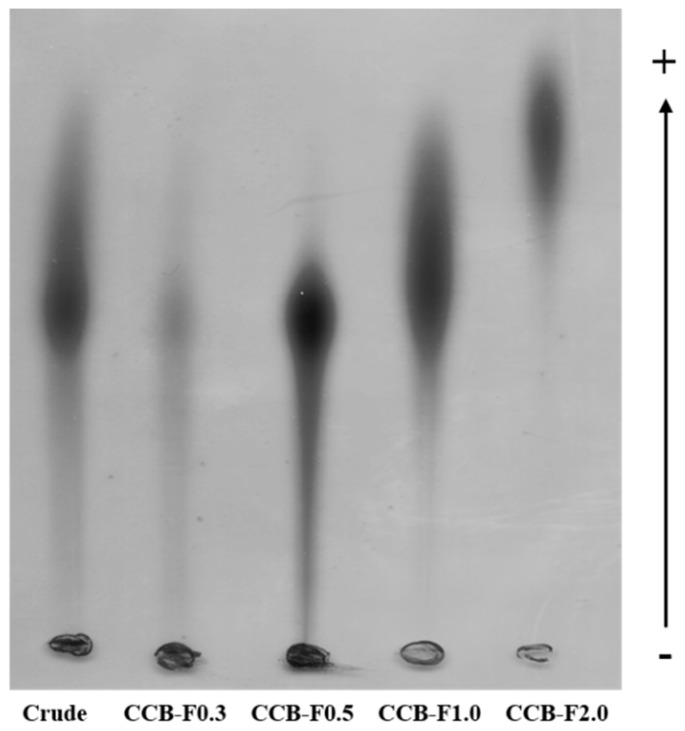
Electrophoretic mobility of the crude extract and polysaccharide fractions from *C. cupressoides* in agarose gel. About 5 µL (50 µg) of each sample were applied in agarose gel prepared in diaminopropane acetate buffer and subjected to electrophoresis, as described in Materials and Methods section.

*C. cupressoides* polysaccharides were subjected to agarose gel electrophoresis. As seen in Figure 1, bands were detected, after toluidine blue staining, that correspond to the predominant SPs population in each of the fractions. In addition, different SPs populations were shown to have different mobilities. The SPs present in CCB-F0.3 and CCB-F0.5 showed similar mobilities, but they were less mobile than the other polysaccharides. CCB-F2.0 SPs had highest mobility, while CCB-F1.0 SPs had an intermediate mobility.

Generally, in agarose gel electrophoresis systems, the mobility of polysaccharides depends on their charge. For example, those with a more negative charge have greater mobility. However, as suggested by Dietrich and Dietrich [25], the use of 1,3-diaminopropane alters this outcome, such that negatively charged polysaccharides are not necessarily more mobile. At the pH used in this experiment (9.0), the SPs assume a conformation in which some sulfate groups are exposed and others not. The exposed sulfate groups react with the diamine, resulting in neutralization of their negative charge. This induces a conformational change in the polysaccharide, causing new sulfate groups to be exposed and subsequently, react with the diamine. This process continues until an equilibrium is reached and the conformation of the polysaccharide no longer changes.

However, the polysaccharide, in this conformational equilibrium condition, will still have sulfate groups that have not reacted with the diamine, and these groups will permit mobility of the polysaccharide during agarose electrophoresis. In sum, the polysaccharide molecules that have the same structure will have the same interaction with the diamine and consequently, will have the same electrophoretic mobility. Meanwhile, structurally different polysaccharides will have different electrophoretic mobilities. The SPs in this study were verified to have similar mobilities to those reported by Costa et al. [23].

### 2.2. Infrared Spectroscopy

**Figure 2:**
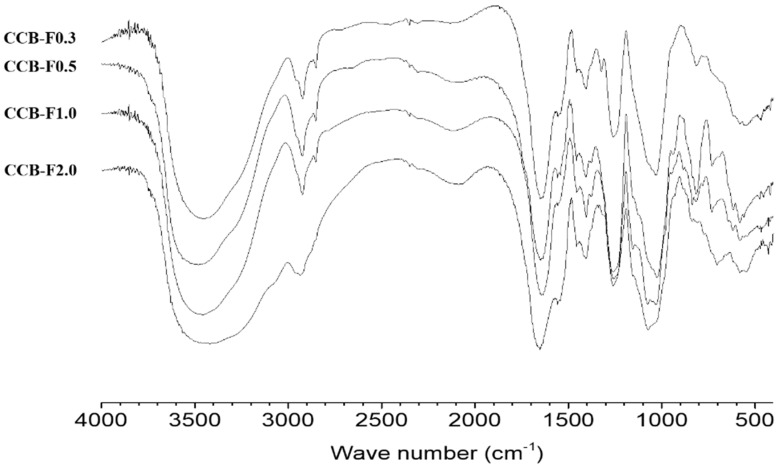
Infrared spectra of sulfated polysaccharide-rich fractions from *C. cupressoides*.

The infrared spectroscopy technique is quick and easy to execute. It is a useful tool that can be used to identify some chemical characteristics from polysaccharides. Infrared spectroscopy analysis detected signals corresponding to SPs functional groups in the four polysaccharide fractions of *C. cupressoides*. As can be seen in Figure 2, bands were identified between 3430–3481 cm^−1^ and 2922–2934 cm^−1^, corresponding to the stretching vibrations O-H and C-H, respectively [16]. Absorption bands in the range of 1641–1655 cm^−1^, corresponding to bound water; 1255–1259 cm^−1^, typical of sulfate groups (S=O); 1030–1075 cm^−1^, corresponding to vibrations of the glycosidic bond of the carbonyl group; and signals around 820 cm^−1^, indicating the presence of asymmetric C-O-S stretching vibrations associated with the C-O-S0_3_ group, were also detected [26,27,28]. The spectra of *C. cupressoides* fractions obtained here were similar to the spectra of the same fractions reported by Costa et al. [23], confirming the structural similarity of the four polysaccharide fractions obtained in the two studies.

The fractions, CCB-F0.3, CCB-F0.5, CCB-F1.0, and CCB-F2.0 and their respective SPs were obtained using the same procedure described by Costa et al. [23]. Furthermore, the fractions obtained here were structurally like those reported by these authors, as seen in both gel electrophoresis and infrared spectra results. Therefore, it may be assumed that the polysaccharides observed here are the same as those obtained by Costa et al. [23]. Therefore, the fractions CCB-F0.3, CCB-F0.5, CCB-F1.0, and CCB-F2.0 and their SPs were evaluated for their immunostimulatory activities.

### 2.3. Cell Viability

**Figure 3:**
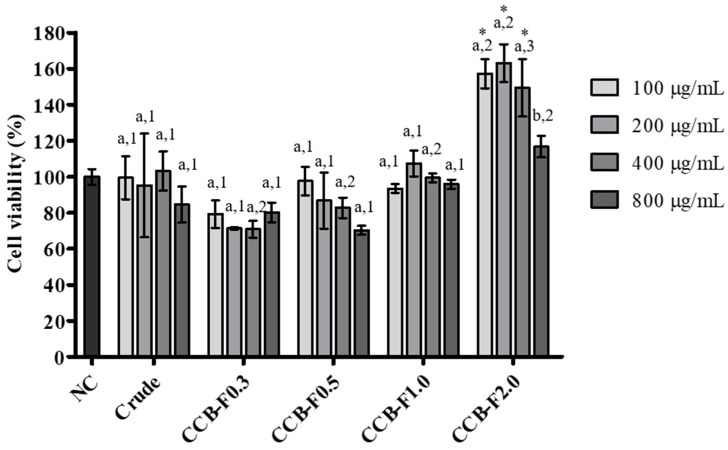
Influence of the crude extract and sulfated polysaccharide-rich fractions from *C. cupressoides* on the viability of RAW 264.7 macrophages. The data presented correspond to means ± standard deviations (*n* = 3). Different letters represent statistically significant differences (*p* < 0.05) between the different concentrations of crude extract or polysaccharide fractions. Different numbers represent statistically significant differences (*p* < 0.05) between the same concentration of the crude extract and polysaccharide fractions. * represents samples that presented statistically significant differences (*p* < 0.05) in relation to the negative control. NC—negative control.

Macrophages are central cellular components of the innate and adaptive immune responses, participating in the production of molecules with microbicidal, tumoricidal, and immunomodulatory functions [29]. Thus, the viability of RAW 264.7 macrophages was evaluated according to 3-(4,5-dimethylthiazolyl-2)-2,5-diphenyltetrazolium bromide (MTT) reduction ability, after treatment with the crude extract and different polysaccharide fractions of *C. cupressoides* (100–800 μg/mL) for 24 h. As shown in Figure 3, in most of the conditions tested, there were no significant changes in MTT reduction ability when compared to the negative control. However, the 100, 200, and 400 μg/mL CCB-F2.0 concentrations showed a significant increase in MTT reduction capacity by up to 60% (*p* < 0.05). These results indicate that *C. cupressoides* SPs had little effect on the viability of RAW 264.7 macrophages and point to a possible absence of cytotoxicity.

### 2.4. NO Production

**Figure 4:**
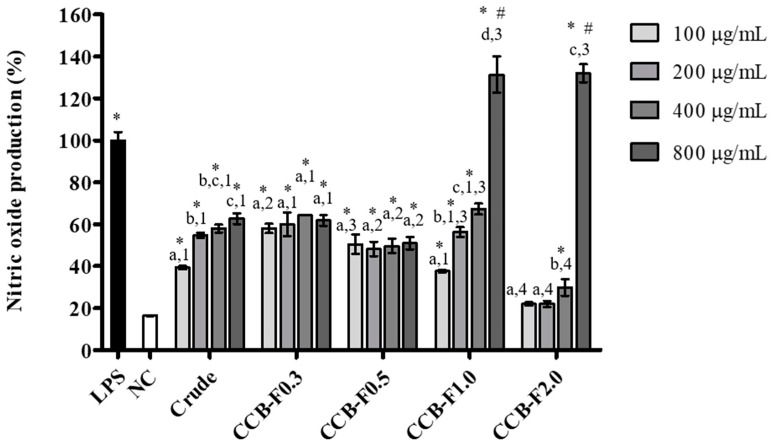
Effect of the crude extract and sulfated polysaccharide-rich fractions from *C. cupressoides* on the NO production. The data presented correspond to means ± standard deviations (*n* = 3). Different letters represent statistically significant differences (*p* < 0.05) between the different concentrations of crude extract or polysaccharide fractions. Different numbers represent statistically significant differences (*p* < 0.05) between the same concentration of the crude extract and polysaccharide fractions. * represents the samples that had a statistically significant difference (*p* < 0.05) in relation to the negative control. # represents statistically significant increases (*p* < 0.01) in NO production relative to the positive control. LPS—Lipopolysaccharide. NC—negative control.

NO is one of the main molecules produced by activated macrophages, with potent cytotoxic effects on pathogenic microorganisms and tumor cells. It also acts as an intracellular messenger in the regulation of several physiological processes [30]. Therefore, the immunomodulatory activity of SPs extracted from seaweeds has been investigated by several authors, through the evaluation of NO levels produced by macrophages [4,14,31]. Therefore, in this study, NO levels were determined in RAW 264.7 macrophages exposed to different concentrations of the crude extract and polysaccharide fractions of *C. cupressoides*. As shown in Figure 4, the crude extract and polysaccharide fractions were able to induce statistically significant increases (*p* < 0.05) in NO production at most concentrations tested, when compared to the negative control. In addition, treatment with 800 μg/mL of CCB-F1.0 or CCB-F2.0 resulted in NO levels that were higher than those produced by LPS-stimulated cells (*p* < 0.01), indicating that these fractions promoted a strong immunostimulatory activity.

SPs are described in the literature as molecules capable of inducing an increase in NO production. In a study by Lee et al. [32], a sulfated galactan obtained from *Codium fragile* was shown to promote an increase in NO production by RAW 264.7 macrophages. Similar immunostimulatory effects are also promoted by SPs from *Dictyopteris divaricate* [33], *Porphyra haitanensis* [14], and *Ecklonia cava* [34] in RAW 264.7 macrophages. The different polysaccharides from *C. cupressoides* induced NO production at different levels. Several studies suggest that the differences in NO production capacity promoted by different SPs types may be related to their chemical characteristics, such as polysaccharide type, molecular weight, monosaccharide composition, conformation and branching of the molecule, and sulfate content [35,36,37]. In this study, the highest NO production values were observed after treatment with the CCB-1.0 and CCB-F2.0 fractions. Costa et al. [23] reported that the most significant differences in the chemical characteristics between these fractions were in the sulfate/total sugars ratio and the molecular weight. In this study, CCB-F1.0 showed a sulfate/total sugars ratio of 0.23, the lowest among the four fractions studied, while CCB-F2.0 had a ratio of 0.53. The molecular weight of SPs in the CCB-F1.0 fraction was 155 kDa and in the CCB-F2.0 fraction, which had the highest molecular weight among all fractions studied, it was 170 kDa.

Studies have observed different immunostimulatory activities of polysaccharides based on their degree of sulfation. In a study of SPs from *Ulva rigida*, Leiro et al. [38] found that NO production was proportional to the amount of sulfate, whereas heterofucans obtained from *Sargassum filipendula*, with similar sugar/sulfate ratios, showed distinct NO production capacities [31]. The authors of this paper suggested that the position in which the sulfate groups are distributed in the molecule seems to be a factor more determining for immunostimulatory capacity than the degree of sulfation. Qi and Kim [36] observed that the reduction in molecular weight of *Chlorella ellipsoidea* SPs resulted in a decrease in NO production. However, carrageenans fractions with lower molecular weight are associated with higher immunostimulatory properties [39]. Nie et al. [40], when evaluating the immunostimulatory capacity of two polysaccharide fractions from stem lettuce, with different sulfate content and molecular weight, observed that macrophages produced the highest quantity of NO when stimulated with the fraction that contained the highest sulfate content and highest molecular weight. Therefore, in view of the heterogeneity and structural diversity of *C. cupressoides* SPs may present some chemical characteristics that individually may not be determinant for its immunostimulatory activity. Due to the higher yield of SPs in the CCB-F1.0 fraction, compared to the CCB-F2.0 fraction (Data not shown), and the absence of a statistically significant difference in NO production capacity, CCB-F1.0 was chosen for the following analyses. In addition, since the largest effect was obtained at 800 μg/mL, this concentration was used in subsequent assays.

### 2.5. Activity and Expression of iNOS

**Figure 5:**
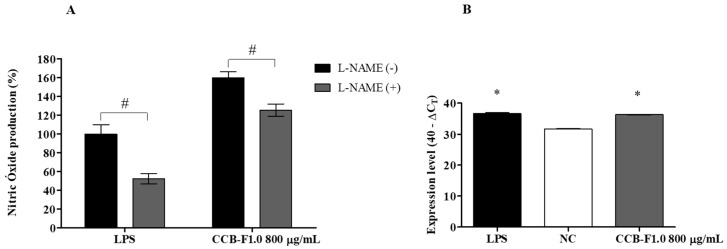
Involvement of iNOS in NO production. (**A**) Percentage of NO production in the presence and absence of the inhibitor, L-NAME. The data presented correspond to means ± standard deviations (*n* = 3). # represents statistically significant differences (*p* < 0.01) between the groups. (**B**) Levels of iNOS mRNA expression. The data presented correspond to means ± standard deviations (*n* = 3). * represents the samples that had a statistically significant difference (*p* < 0.01) in relation to the negative control. LPS—Lipopolysaccharide. NC—negative control.

iNOS is described as the main enzyme involved in the production of NO in activated macrophages [41]. Therefore, experiments were performed to determine if the increase in NO production was related to increased iNOS activity and/or gene expression. Thus, NO levels were determined and the involvement of iNOS was assessed indirectly using its inhibitor, *N*-ω-Nitro-l-arginine methyl ester hydrochloride (L-NAME). Additionally, iNOS gene expression was evaluated by real-time PCR. As can be seen in Figure 5A, L-NAME treatment significantly reduced (*p* < 0.01) NO production in LPS-stimulated cells. Similarly, RAW 264.7 macrophages that were incubated with CCB-F1.0 (800 μg/mL) and L-NAME produced a smaller quantity of NO compared to those stimulated with SPs alone. On the other hand, SPs treatment was shown to increase iNOS gene expression when compared to the negative control, reaching values similar to those observed for macrophages stimulated with LPS (Figure 5B). Increases in transcriptional and translational levels of iNOS have been described as key elements in the immunostimulatory response of macrophages to algal polysaccharide treatment. Qi and Kim [37] found that *C. ellipsoidea* polysaccharides stimulate NO production via increasing the iNOS mRNA expression. In another study, Geng et al. [42] observed that *Saccharina japonica* heteropolysaccharides increase NO levels in RAW 264.7 cells and this effect was accompanied by an increase in iNOS transcription and translation. Similar results were also observed by Borazjani et al. [43], who showed that sulfated polysaccharides from *Sargassum angustifolium* induce NO production by elevating iNOS mRNA levels. Therefore, induction of iNOS gene expression and its catalytic activity appear to be crucial for the activation of and NO production by RAW 264.7 macrophages treated with *C. cupressoides* SPs.

### 2.6. COX-2 Expression

**Figure 6:**
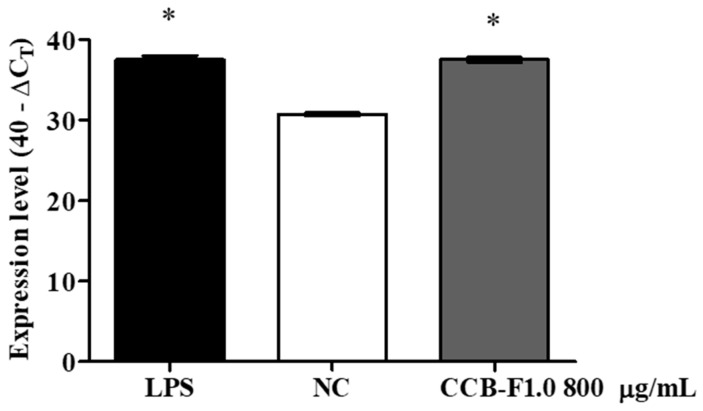
COX-2 expression levels. The data presented correspond to means ± standard deviations (*n* = 3). * represents the samples that had a statistically significant difference (*p* < 0.01) in relation to the negative control. LPS—Lipopolysaccharide. NC—negative control.

Cyclooxygenases are important enzymes involved in the inflammatory response, specifically because they are involved in the synthesis of prostaglandins. SPs obtained from natural sources are described as compounds that stimulate the production of prostaglandins by increasing the expression of the inducible form of cyclooxygenase (COX-2) [20]. Figure 6 shows COX-2 expression levels in RAW 264.7 macrophages stimulated by SPs from the CCB-F1.0 fraction. As can be observed, the treatment with SP increased COX-2 mRNA expression, when compared to the negative control (*p* < 0.01). Leiro et al. [38] reported that the increase in prostaglandin E2 synthesis, induced by *Ulva rigida* SPs, is mainly due to an increase in COX-2 expression. Cao et al. [34] showed that a fucan obtained from *Ecklonia cava* had immunostimulatory properties by increasing COX-2 expression. Similar results have also been observed with SPs from *Agarum cribrosum* [44], which increased COX-2 expression in RAW 264.7 macrophages. Thus, the increase in the COX-2 gene expression by *C. cupressoides* SPs appears to be a fundamental step for its immunostimulatory activity.

### 2.7. Intracellular Reactive Oxygen Species (ROS) Production

**Figure 7:**
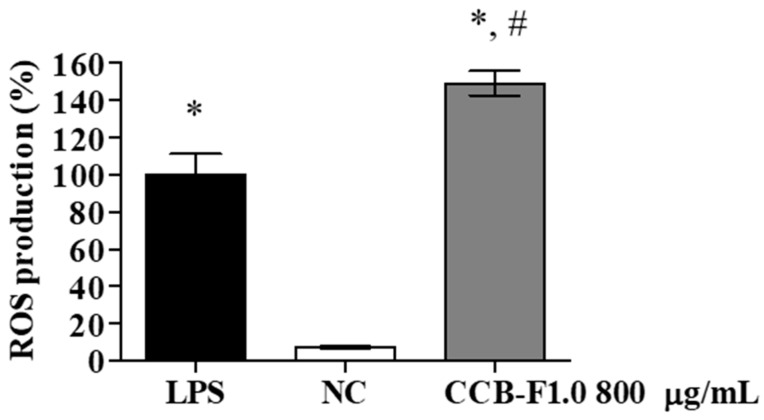
Production of Reactive Oxygen Species (ROS). The data presented correspond to means ± standard deviations (*n* = 3). * represents the samples that had a statistically significant difference (*p* < 0.01) in relation to the negative control. # represents statistically significant increases (*p* < 0.01) in relation to the positive control (LPS). NC—negative control. LPS—Lipopolysaccharide.

In response to proinflammatory agents, activated macrophages increase the production of cytotoxic and microbicidal molecules, such as NO and ROS [45]. Increased ROS production may activate intracellular signaling pathways that promote increased production of inflammatory mediators [46]. As the CCB-F1.0 fraction stimulated an increase in NO levels, its effect on intracellular ROS production in RAW 264.7 macrophages were also assessed. As shown in Figure 7, ROS production in the group treated with the CCB-F1.0 fraction (800 μg/mL) increased 21-fold compared to production in the negative control group. Wang et al. [47] and Jiang et al. [48] have previously shown that the elevation in ROS levels in response to treatment with SPs from the seaweed, *Ascophyllum nodosum*, is an important step in the activation of RAW 264.7 macrophages. Polysaccharides from *U. rigida* are also able to increase ROS production in *Psetta maxima* L. leucocytes. Similarly, different polysaccharide fractions from *Lycium barbarum* have been shown to have important effects on macrophage function through the ROS production [22]. Therefore, the production of intracellular ROS induced by the CCB-1.0 fraction, may play an important role in the cytotoxic capacity of macrophages.

### 2.8. Production of Cytokines

**Figure 8:**
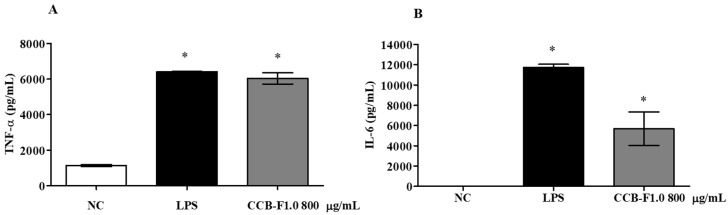
Production of proinflammatory cytokines, TNF-α (**A**) and IL-6 (**B**). The data presented correspond to means ± standard deviations (*n* = 3). * represents a statistically significant difference (*p* < 0.01) in relation to the negative control. LPS—Lipopolysaccharide. NC—negative control.

The presence of pathogenic microorganisms and other tissue-damaging agents may promote the activation of macrophages, resulting in the secretion of cytokines, which play an active role in the eradication of pathogens and in the homeostasis of the immune response [49]. As already described in the literature, SPs may have an important immunostimulatory capacity through the production of cytokines [4,50,51]. Therefore, the effect of CCB-F1.0 on the production of the proinflammatory cytokines, TNF-α and IL-6, was evaluated in RAW 264.7 macrophages. As shown in Figure 8, treatment with SPs promoted a significant increase in the production and secretion of IL-6 and TNF-α, when compared to the group exposed only to culture medium (negative control, *p* < 0.01).

Similar results have also been observed in other studies evaluating the effect of SPs from *S. filipendula* [31], *Spirogyra neglecta* [52], *Hypnea musciformis* [53], *Nemalion helminthoides* [4], *Enteromorpha prolifera* [54], and *Capsosiphon fulvescens* [28] on the production of IL-6 and TNF-α in RAW 264.7 macrophages. Both of these cytokines play important roles in the immune response, as they activate circulating cells and stimulate the production of chemokines and adhesion molecules, thus promoting the recruitment of inflammatory cells to sites of infection [55]. In response to tissue damage, TNF-α is the first proinflammatory cytokine produced [56]. Its participation in the inflammatory response involves inducing the production of other cytokines, such as IL-1, IL-6, and IL-8, in addition to the production of cytotoxic molecules, such as NO and ROS. Furthermore, through its macrophage-activating effect, TNF-α may have potent antitumor activities [57]. IL-6 regulates various cellular functions, including the proliferation and differentiation of T and B lymphocytes and the production of acute-phase proteins in the liver [58]. Therefore, these results suggest that SPs in the CCB-F1.0 fraction may have therapeutic potential due to their effects on the production of cytokines and stimulation of the immune system.

## 3. Materials and Methods

### 3.1. Materials

Agarose (Standart Low-MR) was obtained from BioRad Laboratories (Richmond, CA, USA). 3-(4,5-dimethylthiazolyl-2)-2,5-diphenyltetrazolium bromide (MTT), Griess reagent, *N*-ω-Nitro-l-arginine methyl ester hydrochloride (L-NAME), and 2′,7′-dichlorofluorescin diacetate (DCFH-DA) were purchased from Sigma Chemical Company, (St. Louis, MO, USA). Dulbecco′s modified Eagle′s medium (DMEM) and fetal bovine serum (FBS) were obtained from CULTILAB (Campinas, SP, Brazil). Penicillin and streptomycin were obtained from Gibco (Fort Worth, TX, USA). SYBR Green Master Mix and High-Capacity cDNA Reverse Transcription Kit were from Applied Biosystems (Foster City, CA, USA). The ReliaPrep RNA Cell Miniprep System was purchased from Promega (Madison, WI, USA). DNAse were from Ambion (Life technologies, Carlsbad, CA, USA). Cytokine analysis kits were purchased from BD Biosciences (San Jose, CA, USA). Lipopolysaccharide (LPS, *Escherichia coli* 055: B5) was purchased from Santa Cruz Biotechnology (Dallas, TX, USA). All other solvents and chemical products were of analytical grade.

### 3.2. Seaweed Collection

The green seaweed, *Caulerpa cupressoides* var. *flabellata*, was collected from Nísia Floresta, on the southern coast of the state of Rio Grande do Norte, Brazil (6°1′8.19′′ S and 35°6′33.40′′ W) and then transported to the Laboratório de Biotecnologia de Polímeros Naturais, Departamento de Bioquímica, Universidade Federal do Rio Grande do Norte, RN. Epiphytic species, sediments, and encrusted organisms were removed. After, the seaweeds were dried at 50 °C under ventilation in an oven, ground in a blender and incubated with ethanol to eliminate lipids and pigments. The defatted and depigmented seaweeds were then stored in our laboratory protected from light until the extraction of the polysaccharides. The seaweed was identified by Dr. Valquíria Pereira de Medeiros (Universidade Federal de Juiz de Fora, MG, Brazil).

### 3.3. Extraction and Fractionation of SPs

About 100 g of powdered alga was suspended with five volumes (500 mL) of 0.25 M NaCl and the pH was adjusted to 8.0 with NaOH. Next, 1.5 g of Prolav 750 (Prozyn Biosolutions, São Paulo, SP, Brazil), a mixture of alkaline proteases, was added for proteolytic digestion. After incubation for 18 h at 60 °C, the mixture was filtered through cheesecloth. The resulting extract was referred to as the crude extract and it was then subjected to acetone fractionation, according to the reported method [23]. Briefly, the filtrate was fractionated by precipitation with acetone as follows: 0.3 volumes of ice-cold acetone was added to the solution under gentle agitation and maintained at 4 °C for 24 h. The precipitate formed was collected by centrifugation (10,000× *g*, 20 min), vacuum dried, resuspended in distilled water, and analyzed. The operation was repeated by adding 0.5, 1.0, and 2.0 volumes of acetone to the supernatant. Based on the acetone volumes used in the fractionation step, the obtained fractions were dominated by CCB-F0.3, CCB-F0.5, CCB-F1.0, or CCB-F2.0.

### 3.4. Agarose Gel Electrophoresis in 1,3-diamino Propane Acetate Buffer (PDA)

The electrophoretic mobility of *C. cupressoides* SPs was evaluated by gel electrophoresis in PDA buffer, according to the reported method [25]. Initially, glass slides were coated with 0.6% (*m*/*v*) agarose in PDA buffer (0.05 M, pH 9.0). Subsequently, aliquots of the polysaccharides (about 50 μg) were applied to the gel and subjected to electrophoresis (100 V, 4 °C) for 60 min. After the electrophoretic run, the polysaccharides were precipitated with 0.1% cetyltrimethylammonium bromide (CETAVLON, Sigma Chemical Company, St. Louis, MO, USA) for 2 h at room temperature and the gels were dried using warm air stream. To visualize the SPs, gels were stained with a solution of 0.1% toluidine blue in 1% acetic acid and 50% ethanol. The gel was then de-stained with the same solution without the dye. Three independent analyzes were performed.

### 3.5. Infrared Spectroscopy (FTIR)

To obtain tablets the *C. cupressoides* SPs (5 mg) were mixed thoroughly with dry potassium bromide (KBr). Then, infrared spectra of these tablets were obtained using Fourier Transform Infrared Spectroscopy (IRAffinity-1, Shimadzu Corp., Kyoto, Japan) equipped with IRsolution software (version 1.20, Shimadzu Corp., Kyoto, Japan). The analysis frequency range was 4000 to 400 cm^−1^. Three independent analyses were performed.

### 3.6. Cell Culture

The murine macrophage cell line, RAW 264.7 (ATCC number TIB-71), was cultured in DMEM supplemented with FBS (10% *v*/*v*) and antibiotics (100 U/mL penicillin and 100 μg/mL streptomycin) and maintained in a humidified atmosphere with 5% CO_2_ at 37 °C. The culture medium was changed every three days and upon reaching 80% confluence, cells were subcultured with the aid of a cell scraper.

### 3.7. MTT Reduction Test

To analyze the effect of SPs on cell viability, the ability of RAW 264.7 macrophages to reduce MTT was assessed according to the reported method [59]. Thus, cells were cultivated in 96-well plates at a density of 1 × 10^4^ cells/well. After treatment with different SPs at the different concentrations tested (100–800 μg/mL) for a period of 24 h, the culture medium was substituted for 100 μL of MTT (1 mg/mL dissolved in DMEM) was added. Subsequently, cells were incubated for 4 h in 5% CO_2_ at 37 °C. The wells were then aspirated, and formazan crystals were solubilized by the addition of 100 μL/well of ethanol. Absorbance at 570 nm was measured in an Epoch microplate spectrophotometer from Biotek Instruments Inc. (Winooski, VT, USA). Cell viability was determined in relation to the negative control using the formula: % viability = (A_test_/A_control_) × 100, in which A_test_ corresponds to the absorbance of the experimental group and A_control_, the absorbance of the negative control.

### 3.8. NO Measurement

The immunostimulatory activity of sulfated polysaccharides was determined by quantifying the nitrite levels released in the supernatant of RAW 264.7 macrophages, according to the reported method [60]. Initially, cells were cultured (3 × 10^5^/well) in 24-well plates and exposed to different concentrations of SPs (100–800 μg/mL) for 24 h. LPS (2 μg/mL) was used as a positive control. At the end of the treatment period, 100 μL of supernatant was collected, mixed with 100 μL of Greiss reagent, and incubated for 10 min at room temperature, in the dark. Absorbance at 540 nm was measured in an Epoch microplate spectrophotometer. Sodium nitrite was used as the standard and the results were expressed as a percentage of nitrite production in relation to the positive control (LPS), according to the formula: % nitrite production = (A_test_/A_LPS_) × 100, in which A_test_ corresponds to the absorbance of the experimental group and A_LPS_, the absorbance of LPS (positive control). Additionally, the possible participation of iNOS in NO production was evaluated by quantifying NO levels, as described above, in the presence of the inhibitor, L-NAME (250 μM).

### 3.9. Production of Intracellular ROS

The levels of intracellular ROS were evaluated by quantifying the fluorescence emitted by 2′,7′-dichlorofluorescein, the oxidized form of 2′,7′-dichlorofluorescein diacetate (DCFH-DA). For this purpose, RAW 264.7 macrophages were cultured (3 × 10^5^/well) in 24-well plates and exposed to SPs for 24 h. LPS (2 μg/mL) was used as a positive control. At the end of the treatment period, the supernatant was removed, cells were washed with phosphate buffered saline (PBS) and 100 μM DCFH-DA in DMEM containing 1% FBS were added, and were subsequently incubated at 37 °C for 2 h. DCFH was then removed, cells were washed three times with PBS, and the emitted fluorescence was quantified on a flow cytometer (FACSCanto II; BD Biosciences, Eugene, OR, USA). The results were analyzed in FlowJo software (FlowJo, Ashland, OR, USA) and expressed as percentual (%) of fluorescence emitted relative to LPS.

### 3.10. RNA Extraction and Gene Expression

Gene expression analysis was performed using RAW 264.7 macrophages. Cells (2.5 × 10^6^) were grown in 25 cm^2^ bottles and incubated with SPs at 37 °C for 24 h. Total RNA was extracted using the ReliaPrep^TM^ RNA Cell Miniprep System (Promega Co., Madison, WI, USA), according to the manufacturer’s instructions and quantified in a NanoDrop One instrument (Thermo Fisher Scientific, Waltham, MA, USA). After extraction of the RNA, the total RNA was treated with DNAse according to manufacturer′s instruction. Then, the complementary DNA was synthesized using a High Capacity cDNA Reverse Transcription kit (Applied Biosystems, Foster City, CA, USA), according to the manufacturer′s instructions. Amplification reactions were performed using SYBR Green PCR Master Max, as instructed by the manufacturer, on a QuantStudio 5 Real-Time PCR System (Applied Biosystems). The experimental design involved three biological replicates and three technical replicates for each reaction.

The following PCR primers were used: COX-2, 5′-CTGGAACATGGACTCACTCAGTTG-3′ (Forward), 5-′AGGCCTTTGCCACTGCTTG-3′(Reverse); iNOS, 5′-ACCTTGTTCAGCTCAGCCTTCAAC-3′ (Forward), 5′-GTGCTTGTCACCACCAGCAGTAGT-3′ (Reverse); β-actin, 5′-TCATGAAGTGTGCGTTGACATCCGT-3′ (Forward), 5′-CCTAGAAGC ATTTGCGGTGCACGAG-3′ (Reverse). Gene expression was normalized by β-actin (reference gene). The efficiency of PCR (E) was calculated using LinRegPCR software, according to the reported method [61]. Threshold cycles (Cts) were normalized to an efficiency equal to 2 [Ct′ = Ct × (log2 E/log2 2)] and relative expression was calculated according to the method of Pant et al. [62].

### 3.11. Cytokine Production

The supernatant of cells exposed to SPs in the experiment used for RNA extraction was also used for quantification of the cytokines, IL-6 and TNF-α. Cytokines were quantified using the BD Cytometric Bread Array (CBA) Mouse Th1/Th2/Th17 Cytokine Kit (BD Biosciences, Franklin Lakes, NJ, USA), according to the manufacturer′s guidelines, on a FACSCanto II flow cytometer.

### 3.12. Statistical Analysis

Statistical analysis was performed with Prism 5.00 (GraphPad, San Diego, CA, USA). Results were expressed as mean ± standard deviation. Statistical differences between groups were assessed using analysis of variance and the Student Newman–Keuls test. Values of *p* < 0.05 were considered statistically significant.

## 4. Conclusions

Four SPs-rich fractions were obtained from *C. cupressoides* after proteolysis and acetone precipitation. Electrophoretic and FTIR analyses confirmed that these fractions contain the same SPs described in a previous report by our group. The crude extract and the sulfated polysaccharide-rich fractions had SPs with different immunostimulatory abilities, but they did not have a considerable effect on the viability of RAW 264.7 macrophages. In particular, the CCB-F1.0 fraction showed a remarkable ability to stimulate NO production and therefore, additional analyses were performed to better understand its immunostimulatory potential. In these analyses, it was found that NO production was dependent on the activity and expression of iNOS. COX-2 mRNA levels were also found to increase after exposure to these SPs. Finally, treatment with SPs of the CCB-F1.0 fraction induced a significant increase in the production of other inflammatory mediators, such as ROS and the cytokines, TNF-α and IL-6. Together, these results showed that SPs from *C. cupressoides* have a potent immunostimulatory capacity on RAW 264.7 macrophages and therefore, have potential application in pharmacological and functional food products.

## References

[1] Sanjeewa K.K.A., Jeon Y. (2018). Edible brown seaweeds: A review. J. Food Bioact..

[2] Wijesinghe W.A.J.P., Jeon Y.-J. (2012). Biological activities and potential industrial applications of fucose rich sulfated polysaccharides and fucoidans isolated from brown seaweeds: A review. Carbohydr. Polym..

[3] Deniaud-Bouët E., Hardouin K., Potin P., Kloareg B., Hervé C. (2017). A review about brown algal cell walls and fucose-containing sulfated polysaccharides: Cell wall context, biomedical properties and key research challenges. Carbohydr. Polym..

[4] Pérez-recalde M., Matulewicz M.C., Pujol C.A., Carlucci M.J. (2014). In vitro and in vivo immunomodulatory activity of sulfated polysaccharides from red seaweed *Nemalion helminthoides*. Int. J. Biol. Macromol..

[5] Ngo D., Kim S. (2013). Sulfated polysaccharides as bioactive agents from marine algae. Int. J. Biol. Macromol..

[6] De Jesus Raposo M.F., De Morais A.M.B., De Morais R.M.S.C. (2015). Marine polysaccharides from algae with potential biomedical applications. Mar. Drugs.

[7] Wang L., Wang X., Wu H., Liu R. (2014). Overview on biological activities and molecular characteristics of sulfated polysaccharides from marine green algae in recent years. Mar. Drugs.

[8] Surayot U., You S.G. (2017). Structural effects of sulfated polysaccharides from *Codium fragile* on NK cell activation and cytotoxicity. Int. J. Biol. Macromol..

[9] Berri M., Olivier M., Holbert S., Dupont J., Demais H., Le Goff M., Collen P.N. (2017). Ulvan from *Ulva armoricana* (Chlorophyta) activates the PI3K/Akt signalling pathway via TLR4 to induce intestinal cytokine production. Algal Res..

[10] Li C., Huang Q., Fu X., Yue X.J., Liu R.H., You L.J. (2015). Characterization, antioxidant and immunomodulatory activities of polysaccharides from *Prunella vulgaris* Linn. Int. J. Biol. Macromol..

[11] Zhang Y., Zhang M., Jiang Y., Li X., He Y., Zeng P., Guo Z., Chang Y., Luo H., Liu Y. (2018). Lentinan as an immunotherapeutic for treating lung cancer: A review of 12 years clinical studies in China. J. Cancer Res. Clin. Oncol..

[12] Guo Z., Hu Y., Wang D., Ma X., Zhao X., Zhao B., Wang J., Liu P. (2009). Sulfated modification can enhance the adjuvanticity of lentinan and improve the immune effect of ND vaccine. Vaccine.

[13] Tiwari R., Latheef S.K., Ahmed I., Iqbal H.M.N., Bule M.H., Dhama K., Samad H.A., Karthik K., Alagawany M., El-Hack M.E.A. (2018). Herbal immunomodulators, a remedial panacea for the designing and developing effective drugs and medicines: Current scenario and future prospects. Curr. Drug Metab..

[14] Liu Q.M., Xu S.S., Li L., Pan T.M., Shi C.L., Liu H., Cao M.J., Su W.J., Liu G.M. (2017). In vitro and in vivo immunomodulatory activity of sulfated polysaccharide from *Porphyra haitanensis*. Carbohydr. Polym..

[15] Bi D., Yu B., Han Q., Lu J., White W.L., Lai Q., Cai N., Luo W., Gu L., Li S. (2018). Immune activation of RAW264.7 macrophages by low molecular weight fucoidan extracted from New Zealand *Undaria pinnatifida*. J. Agric. Food Chem..

[16] Yang Y., Xing R., Liu S., Qin Y., Li K., Yu H., Li P. (2018). Immunostimulatory effects of sulfated chitosans on RAW 264.7 mouse macrophages via the activation of PI3 K/Akt signaling pathway. Int. J. Biol. Macromol..

[17] Makarenkova I.D., Akhmatova N.K., Ermakova S.P., Besednova N.N. (2017). Morphofunctional changes of dendritic cells induced by sulfated polysaccharides of brown algae. Biochem. (Moscow) Suppl. Ser. B Biomed. Chem..

[18] Wu F., Zhou C., Zhou D., Ou S., Liu Z., Huang H. (2018). Immune-enhancing activities of chondroitin sulfate in murine macrophage RAW 264.7 cells. Carbohydr. Polym..

[19] Di T., Chen G., Sun Y., Ou S., Zeng X., Ye H. (2017). Antioxidant and immunostimulating activities in vitro of sulfated polysaccharides isolated from *Gracilaria rubra*. J. Funct. Foods.

[20] Sanjeewa K.K.A., Fernando I.P.S., Kim E.A., Ahn G., Jee Y., Jeon Y.J. (2017). Anti-inflammatory activity of a sulfated polysaccharide isolated from an enzymatic digest of brown seaweed *Sargassum horneri* in RAW 264.7 cells. Nutr. Res. Pract..

[21] Jose G.M., Kurup G.M. (2017). The efficacy of sulfated polysaccharides from *Padina tetrastromatica* in modulating the immune functions of RAW 264.7 cells. Biomed. Pharmacother..

[22] Deng X., Liu Q., Fu Y., Luo X., Hu M., Ma F., Wang Q., Lai X., Zhou L. (2018). Effects of *Lycium barbarum* polysaccharides with different molecular weights on function of RAW264.7 macrophages. Food Agric. Immunol..

[23] Costa M.S.S.P., Costa L.S., Cordeiro S.L., Almeida-Lima J., Dantas-Santos N., Magalhães K.D., Sabry D.A., Albuquerque I.R.L., Pereira M.R., Leite E.L. (2012). Evaluating the possible anticoagulant and antioxidant effects of sulfated polysaccharides from the tropical green alga *Caulerpa cupressoides* var. *flabellata*. J. Appl. Phycol..

[24] Costa L.S., Fidelis G.P., Cordeiro S.L., Oliveira R.M., Sabry D.A., Câmara R.B.G., Nobre L.T.D.B., Costa M.S.S.P., Almeida-Lima J., Farias E.H.C. (2010). Biological activities of sulfated polysaccharides from tropical seaweeds. Biomed. Pharmacother..

[25] Dietrich C.P., Dietrich S.M.C. (1976). Electrophoretic behaviour of acidic mucopolysaccharides in diamine buffers. Anal. Biochem..

[26] Sudharsan S., Subhapradha N., Seedevi P., Shanmugam V., Madeswaran P., Shanmugam A., Srinivasan A. (2015). Antioxidant and anticoagulant activity of sulfated polysaccharide from *Gracilaria debilis* (Forsskal). Int. J. Biol. Macromol..

[27] Wang X., Wang J., Zhang J., Zhao B., Yao J., Wang Y. (2010). Structure-antioxidant relationships of sulfated galactomannan from guar gum. Int. J. Biol. Macromol..

[28] Na Y.S., Kim W.J., Kim S.M., Park J.K., Lee S.M., Kim S.O., Synytsya A., Park Y.I. (2010). Purification, characterization and immunostimulating activity of water-soluble polysaccharide isolated from *Capsosiphon fulvescens*. Int. Immunopharmacol..

[29] Schultze J.L., Schmidt S.V. (2016). Molecular features of macrophage activation. Semin. Immunol..

[30] Bogdan C. (2015). Nitric oxide synthase in innate and adaptive immunity: An update. Trends Immunol..

[31] Telles C.B.S., Mendes-Aguiar C., Fidelis G.P., Frasson A.P., Pereira W.O., Scortecci K.C., Camara R.B.G., Nobre L.T.D.B., Costa L.S., Tasca T. (2018). Immunomodulatory effects and antimicrobial activity of heterofucans from *Sargassum filipendula*. J. Appl. Phycol..

[32] Lee J.B., Ohta Y., Hayashi K., Hayashi T. (2010). Immunostimulating effects of a sulfated galactan from *Codium fragile*. Carbohydr. Res..

[33] Cui Y., Liu X., Li S., Hao L., Du J., Gao D.H., Kang Q., Lu J. (2018). Extraction, characterization and biological activity of sulfated polysaccharides from seaweed *Dictyopteris divaricata*. Int. J. Biol. Macromol..

[34] Cao R.A., Lee Y.J., You S.G. (2014). Water soluble sulfated-fucans with immune-enhancing properties from *Ecklonia cava*. Int. J. Biol. Macromol..

[35] Ferreira S.S., Passos C.P., Madureira P., Vilanova M., Coimbra M.A. (2015). Structure-function relationships of immunostimulatory polysaccharides: A review. Carbohydr. Polym..

[36] Qi J., Kim S.M. (2018). Effects of the molecular weight and protein and sulfate content of *Chlorella ellipsoidea* polysaccharides on their immunomodulatory activity. Int. J. Biol. Macromol..

[37] Qi J., Kim S.M. (2017). Characterization and immunomodulatory activities of polysaccharides extracted from green alga *Chlorella ellipsoidea*. Int. J. Biol. Macromol..

[38] Leiro J.M., Castro R., Arranz J.A., Lamas J. (2007). Immunomodulating activities of acidic sulphated polysaccharides obtained from the seaweed *Ulva rigida* C. Agardh. Int. Immunopharmacol..

[39] Stephanie B., Eric D., Sophie F.M., Christian B., Yu G. (2010). Carrageenan from *Solieria chordalis* (Gigartinales): Structural analysis and immunological activities of the low molecular weight fractions. Carbohydr. Polym..

[40] Nie C., Zhu P., Ma S., Wang M., Hu Y. (2018). Purification, characterization and immunomodulatory activity of polysaccharides from stem lettuce. Carbohydr. Polym..

[41] Uehara E.U., Shida B.D.S., de Brito C.A. (2015). Role of nitric oxide in immune responses against viruses: Beyond microbicidal activity. Inflamm. Res..

[42] Geng L., Hu W., Liu Y., Wang J., Zhang Q. (2018). A heteropolysaccharide from *Saccharina japonica* with immunomodulatory effect on RAW 264.7 cells. Carbohydr. Polym..

[43] Borazjani N.J., Tabarsa M., You S., Rezaei M. (2018). Purification, molecular properties, structural characterization, and immunomodulatory activities of water soluble polysaccharides from *Sargassum angustifolium*. Int. J. Biol. Macromol..

[44] Cho M., Lee D.J., Kim J.K., You S. (2014). Molecular characterization and immunomodulatory activity of sulfated fucans from *Agarum cribrosum*. Carbohydr. Polym..

[45] Lee J.S., Kwon D.S., Lee K.R., Park J.M., Ha S.J., Hong E.K. (2015). Mechanism of macrophage activation induced by polysaccharide from *Cordyceps militaris* culture broth. Carbohydr. Polym..

[46] Wang W., Zou Y., Li Q., Mao R., Shao X., Jin D., Zheng D., Zhao T., Zhu H., Zhang L. (2016). Immunomodulatory effects of a polysaccharide purified from *Lepidium meyenii* Walp. on macrophages. Process Biochem..

[47] Wang Y., Jiang Z., Kim D., Ueno M., Okimura T., Yamaguchi K., Oda T. (2013). Stimulatory effect of the sulfated polysaccharide ascophyllan on the respiratory burst in RAW264.7 macrophages. Int. J. Biol. Macromol..

[48] Jiang Z., Ueno M., Nishiguchi T., Abu R., Isaka S., Okimura T., Yamaguchi K., Oda T. (2013). Importance of sulfate groups for the macrophage-stimulating activities of ascophyllan isolated from the brown alga in *Ascophyllum nodosum*. Carbohydr. Res..

[49] Shapouri-Moghaddam A., Mohammadian S., Vazini H., Taghadosi M., Esmaeili S.A., Mardani F., Seifi B., Mohammadi A., Afshari J.T., Sahebkar A. (2018). Macrophage plasticity, polarization, and function in health and disease. J. Cell. Physiol..

[50] Tabarsa M., You S.G., Dabaghian E.H., Surayot U. (2018). Water-soluble polysaccharides from *Ulva intestinalis*: Molecular properties, structural elucidation and immunomodulatory activities. J. Food Drug Anal..

[51] Surayot U., Lee J.H., Kanongnuch C., Peerapornpisal Y., Park W.J., You S.G. (2016). Structural characterization of sulfated arabinans extracted from *Cladophora glomerata* Kützing and their macrophage activation. Biosci. Biotechnol. Biochem..

[52] Surayot U., Wang J.G., Lee J.H., Kanongnuch C., Peerapornpisal Y., You S.G. (2015). Characterization and immunomodulatory activities of polysaccharides from *Spirogyra neglecta* (Hassall) Kützing. Biosci. Biotechnol. Biochem..

[53] Alves M.G.C.F., Almeida-Lima J., Paiva A.A.O., Leite E.L., Rocha H.A.O. (2016). Extraction process optimization of sulfated galactan-rich fractions from *Hypnea musciformis* in order to obtain antioxidant, anticoagulant, or immunomodulatory polysaccharides. J. Appl. Phycol..

[54] Kim J.K., Cho M.L., Karnjanapratum S., Shin I.S., You S.G. (2011). In vitro and in vivo immunomodulatory activity of sulfated polysaccharides from *Enteromorpha prolifera*. Int. J. Biol. Macromol..

[55] Duque G.A., Descoteaux A. (2014). Macrophage cytokines: Involvement in immunity and infectious diseases. Front. Immunol..

[56] Ribeiro V.P., Arruda C., Abd El-Salam M., Bastos J.K. (2018). Brazilian medicinal plants with corroborated anti-inflammatory activities: A review. Pharm. Biol..

[57] Bertazza L., Mocellin S. (2010). The dual role of tumor necrosis factor (TNF) in cancer biology. Curr. Med. Chem..

[58] Xu Y., Zhang Y., Ye J. (2018). IL-6: A potential role in cardiac metabolic homeostasis. Int. J. Mol. Sci..

[59] Mosmann T. (1983). Rapid colorimetric assay for cellular growth and survival: Application to proliferation and cytotoxicity assays. J. Immunol. Methods.

[60] Green L.C., Wagner D.A., Glogowski J., Skipper P.L., Wishnok J.S., Tannenbaum S.R. (1982). Analysis of nitrate, nitrite, and [15 N] nitrate in biological fluids. Anal. Biochem..

[61] Ramakers C., Ruijter J.M., Lekanne Deprez R.H., Moorman A.F.M. (2003). Assumption-free analysis of quantitative real-time polymerase chain reaction (PCR) data. Neurosci. Lett..

[62] Pant B.D., Buhtz A., Kehr J., Scheible W.R. (2008). MicroRNA399 is a long-distance signal for the regulation of plant phosphate homeostasis. Plant J..

